# Exploring the caregiving perceptions and needs of family caregivers of older stroke patients: a descriptive qualitative study

**DOI:** 10.1080/17482631.2025.2588908

**Published:** 2025-11-25

**Authors:** Xuejuan Yang, Haiyan Wang, Siying Chen, Yin He, Yuying Chen

**Affiliations:** a School of Nursing, Kunming Medical University, Kunming, Yunnan, People's Republic of China; b Department of Neurology, The Second Affiliated Hospital of Kunming Medical University, Kunming, Yunnan, People's Republic of China; c Department of Radiology, Yunnan Geriatric Hospital, Kunming, Yunnan, People's Republic of China

**Keywords:** Old age, acute ischemic strokes, caregiver, qualitative research, nursing

## Abstract

**Background:**

Stroke is a leading cause of death and disability globally, with high incidence in aging Asian populations. Elderly stroke survivors often have physiological dysfunctions, relying heavily on primary informal caregivers. However, Chinese caregivers face unique cultural and practical challenges (e.g., close bonding, filial piety-driven self-sacrifice, urban-rural medical insurance gaps) has not been explored from a qualitative perspective.

**Purpose:**

This study aimed to explore the caregiving experiences, emotional states, and unmet needs of primary caregivers of elderly stroke patients in China, using Maslow’s Hierarchy of Needs as a theoretical framework to guide analysis and identify targeted support strategies.

**Methods:**

A descriptive qualitative study was conducted from March to April 2024 in the neurology department of a tertiary Grade-A hospital in southwestern China. Purposive sampling with maximum variation was used to recruit 19 primary caregivers.Data were collected via 30–45 minute semi-structured one-on-one interviews and analyzed using directed content analysis with NVivo software.

**Results:**

Three core themes emerged: 1) Caregiving challenges (physical/mental exhaustion, economic pressure, role adaptation difficulties, lifestyle disruption); 2) Caregiver growth (enhanced mental resilience, improved health awareness, strengthened family bonds, reshaped life meaning); 3) Diverse support needs (instrumental, informational, emotional, social support). These themes mapped to all tiers of Maslow’s Hierarchy of Needs.

**Conclusions:**

Chinese caregivers of elderly stroke patients face multi-dimensional burdens but also experience personal growth. Healthcare professionals should use Maslow’s theory to provide comprehensive support to improve caregivers’ well-being and long-term care quality.

## Plain language summary

**What is known?**

- Caregivers are under tremendous physical and emotional stress.
- Caregivers are in desperate need of social assistance to alleviate feelings of loneliness and powerlessness.
- Nurses play a vital role in reducing caregivers’ stress and enhancing their capacity for care.

**What is new?**

- **Cultural and Contextual Insights on Caregiving in Mainland China:** This study offers an in-depth exploration of the emotional journey and unmet needs of primary caregivers of elderly stroke survivors within the unique socio-cultural context of mainland China, highlighting the impact of filial piety norms and healthcare system characteristics.
- **Human-Centered Care Paradigm in a Chinese Context:** By emphasizing the interdependence of patient outcomes and caregiver well-being within familial and cultural expectations, this research advocates for an integrated care model tailored to the realities of Chinese family structures and support systems.
- **Policy and Practice Implications for China:** Findings underscore the urgency of developing culturally and systemically appropriate support mechanisms (e.g., respite care, psychological counseling, community resources) within mainland China to alleviate caregiver burden and improve long-term care sustainability.

## 1 Introduction

Stroke is a severe neurological disorder that has emerged as a global public health challenge and remains one of the leading causes of death and disability worldwide.Research indicates that the incidence of stroke is rising in most Asian countries, characterized by earlier onset ages and a higher proportion of ischemic stroke than in Western nations (Mehndiratta et al., 2014). In China, it is a major non-communicable disease that threatens national health, with over two million new cases annually (Krishnamurthi et al., 2020; Wu et al., 2019). Given the clinical predominance and long-term care demands associated with ischemic stroke, investigating caregivers of this specific patient population is imperative to understand their distinct support needs.Elderly stroke survivors often experience physiological dysfunction (e.g., hemiplegia, aphasia, cognitive impairment), underscoring the critical role of primary caregivers in their treatment and rehabilitation (Stinear et al., 2020). As the aging population grows, so does the demand for informal care (Zarzycki & Morrison, 2021), imposing substantial burdens on patients, families, and society (Krishnamurthi et al., 2020; Sharrief & Grotta, 2019).

Primary caregivers, defined as informal caregivers (typically family members or friends), provide care beyond that provided by conventional medical services. Caregivers assume multi-faceted roles including daily living assistance, emotional support, and rehabilitation coordination. Studies have revealed that caregiving often strains family relationships, leading to conflicts between patients and caregivers (McCarthy et al., 2020). Additionally, caregivers are at an elevated risk of depression, anxiety, and reduced quality of life during caregiving stress (Loh et al., 2017; Saban et al., 2010). Collectively, caregiver burden stems from four primary domains: physical health, social function, economic strain, and mental well-being (Tziaka et al., 2024).

Existing research on stroke caregivers has documented these challenges across various cultural settings. However, within mainland China, caregiving experiences are profoundly shaped by unique sociocultural factors, including Confucian norms of filial piety, reliance on family-based rather than institutional care, and disparities in healthcare access between urban and rural regions (Gabriel et al., 2022; Qiu et al., 2018). For instance, Chinese primary family caregivers reported nearly three times higher odds of perceived burden than non-Chinese counterparts, often prioritizing self-sacrifice and enduring significant emotional and financial strain without seeking external support (Gabriel et al., 2022). A qualitative study in Singapore further highlighted that adult child caregivers sought external help more frequently than spousal caregivers, suggesting that cultural expectations around familial duty may intensify burden for certain caregiver groups (Tyagi et al., 2023).

Despite these challenges, caregiving can yield positive psychological outcomes that mitigate burden’s adverse effects. For example, Mei et al. (2018), via structured interviews with 145 Zhengzhou-based stroke caregivers in China, found stronger positive meaning-finding correlates with lower caregiving burden and better mental health, while heavier burden links to more severe anxiety/depression.Notably, benefit finding did not moderate the burden-well-being link but acted as a key mediator—higher burden reduced benefit finding, which in turn worsened mental health. This means caregivers who identified caregiving benefits still had better well-being despite heavy burden, with clinical relevance: nurses could improve caregivers’ mental health by guiding them to recognize such benefits.Similar protective effects have been observed in other Asian contexts; for instance, Chaknum et al. (2023) reported that adaptive coping and strong family functioning reduced burden among Thai stroke caregivers.

Nevertheless, prior studies have not sufficiently explored how the multifaceted needs of Chinese caregivers align with a structured theoretical framework. While qualitative research has described burdens and coping strategies, there remains a lack of analysis linking caregivers’ experiences to a hierarchical model of human needs, which could inform targeted support interventions. Maslow’s Hierarchy of Needs (Zhang et al., 2022) offers a comprehensive framework for understanding how basic needs must be met before higher-level psychological growth can occur. This theory is particularly relevant in the Chinese caregiving context, where cultural values (e.g., prioritizing family obligations over self-care) may disrupt the natural progression of need fulfillment. For instance, caregivers often neglect their own physiological and safety needs due to filial piety expectations, thereby impeding their capacity for achieving self-esteem or self-actualization through caregiving. Applying Maslow’s model allows for a systematic identification of unmet needs across different levels (e.g., lack of respite care compromising physiological needs; financial insecurity undermining safety needs) and clarifies how cultural and systemic factors exacerbate these gaps. Moreover, it provides a culturally sensitive lens to explore how caregivers negotiate between culturally rooted caregiving roles shaped by Chinese traditions (such as filial piety and family-centric obligations) and personal well-being, offering insights distinct from those provided by other caregiving models that focus solely on stress or burden. Thus, this study utilizes Maslow’s framework to: ① map the experiences of caregivers of elderly ischemic stroke patients in mainland China across all levels of need; ② identify structural and cultural barriers to need fulfillment; and ③ propose targeted interventions that address these needs in a hierarchical manner, ultimately enhancing both caregiver well-being and care quality.

## 2 Methods

### 2.1 Study design

This study adopted a descriptive qualitative research approach, a methodological framework focused on understanding how individuals subjectively construct meaning from their experiences within specific cultural, social, and contextual settings. This approach was selected because the study aimed to explore the caregiving experiences, emotional states, and unmet needs of primary caregivers of elderly stroke patients in China—where cultural values (e.g., filial piety, family-centric care) significantly shape caregivers’ perceptions and behaviors. Descriptive qualitative research aligns with this goal by prioritizing participants’ unique, lived perspectives over pre-determined assumptions, and it guided key decisions across the research process to ensure rigor: for instance, it informed the use of semi-structured interviews (to deeply elicit subjective experiences rather than restrict responses to fixed questions) and directed content analysis (which balances theoretical guidance from Maslow’s Hierarchy of Needs with openness to emergent themes reflecting caregivers’ real-life realities).

This study was conducted at a hospital in Kunming, China, from March to April 2024. Data were collected via 30–45 minute semi-structured one-on-one interviews, focusing on caregivers’ caregiving routines, emotional experiences, and support needs. Directed content analysis was used to interpret data from 19 respondents, with iterative refinement of codes to ensure alignment with participants’ subjective accounts.

### 2.2 Participants and settings

To explore contextual caregiving experiences of primary caregivers for elderly ischemic stroke patients in China, this study used purposive sampling (with maximum diversity sampling) to capture multidimensional perspectives.Maximum variation sampling stratified participants by three dimensions: ① Gender (11 males, 8 females); ② Education (elementary school to master’s degree); ③ Perceived caregiving burden (light vs. heavy). Recruitment was conducted in the neurology department of a Class A tertiary hospital (a regional stroke center), ensuring the sample aligned with the Chinese cultural context for representativeness.

Recruitment Procedures: Recruitment was led by 2 geriatric nursing master’s students (direct recruiters) and a neurology head nurse (supervisor). From March to April 2024, during daily ward rounds, the interview-leading master’s student (positioned as a “supportive researcher” to avoid power imbalances) first confirmed potential caregivers’ roles with patients/ward staff, then introduced the study in private spaces (e.g., consultation rooms). Eligible candidates received an Ethics Committee-approved recruitment flyer (detailing study focus, 30–45 minute interview, contact info) and 24–48 hours to consider. Interested caregivers had follow-up meetings to clarify objectives/procedures, address questions, and confirm eligibility—this two-step process ensured informed consent and trust.

The study defined clear inclusion and exclusion criteria for participants: inclusion criteria included ① Being the patient's spouse or relative; ② Age ≥ 18 years with daily caregiving duration ≥4 hours (to define “primary caregiver”); ③ No cognitive impairment and possessing basic literacy and communication skills; ④ Voluntary participation and signed informed consent. Exclusion criteria included: ① Experiencing major stressful events within the past 3 months; ② Working in healthcare-related professions (to avoid bias).

Sample size followed qualitative data saturation, with three operational criteria: ① No new codes/subthemes in 3 consecutive interviews; ② Core theme frequency stabilization (new illustrative quotes per theme dropped by ≥80% vs. initial 5 interviews); ③ ≥90% intercoder agreement (Cohen’s Kappa). Saturation was reached after 15 interviews; 4 additional caregivers were recruited to verify theme stability (no deviations), resulting in 19 participants. Eligibility screening was done by geriatric nursing master’s students.

### 2.3 Data collection

#### 2.3.1 Interview guide development​

To develop the semi-structured interview guide, a three-step process was implemented, grounded in literature, theory, and contextual feedback:​

Literature and Theoretical Foundation: A systematic review of literature on stroke caregiver experiences was conducted (Akgun-Citak et al., 2020; Li et al., 2023; Pallerla et al., 2024), identifying core domains (caregiving burden, social support, unmet needs) that aligned with the study’s constructivist focus on contextual meaning-making and Maslow’s Hierarchy of Needs. The latter was used to frame questions addressing basic care needs (e.g., daily routine management), emotional safety (e.g., emotional experiences), and self-actualization (e.g., perceived rewards of caregiving).​

Research Group Consultation: The research team (a Gerontological nursing nursing instructor, neurology head nurse, and two master’s candidates in nursing) refined the guide to reflect southwestern China’s cultural context—for example, adding questions about “filial piety-related care pressures” to capture region-specific caregiving norms.​

Preliminary Interview Validation: Three preliminary interviews were conducted with caregivers meeting inclusion criteria. Feedback indicated ambiguity in questions about “community resource support”; thus, revisions were made to specify “community-based home care services” and “post-discharge rehabilitation guidance.” An additional follow-up question (“How do these challenges affect your willingness to continue caregiving?”) was added to explore emotional impacts in depth.​

The finalized interview questions are as follows: ① How do you manage the patient’s daily care routines (e.g., feeding, bathing, medication adherence)? ② Can you describe your emotional experiences during caregiving (e.g., anxiety, guilt, relief)? ③ How has caregiving impacted your work, daily life, and family relationships (e.g., work absences, reduced time with family members)? ④ What challenges or rewards have you encountered in caregiving, and how did you cope with the challenges? ⑤ What support have you received from family, healthcare providers, or community resources (e.g., family assistance with care, hospital follow-up calls, community day care services)? ⑥ What unmet needs do you identify in your caregiving role (e.g., lack of professional care training, insufficient emotional counseling)? ⑦ How could healthcare professionals better support you in your caregiving role?​

#### 2.3.2 Iterative data collection process​

Data collection (March–April 2024) and analysis were conducted concurrently to enable iterative adjustments, as follows:​

Preliminary Analysis Checkpoints: After every 3 interviews, the research team (interviewer, neurology head nurse, Gerontological nursing nursing instructor) held meetings to review preliminary coding results (e.g., initial codes of “insufficient post-discharge rehabilitation guidance” and “caregiver guilt about neglecting personal health”).​

Adjustments to Data Collection: Deepening Exploration of Emerging Needs: Preliminary analysis showed 80% of caregivers reported “confusion about stroke recurrence prevention”; a follow-up question (“What information about stroke recurrence prevention do you hope to obtain from healthcare providers?”) was added to address this unmet need.​

Capturing Diverse Perspectives: To confirm and disconfirm themes related to “family support,” 2 additional caregivers with no family assistance were recruited to compare their experiences with those who received family support, minimizing bias toward “family-centric care” norms.​

#### 2.3.3 Interview implementation​

Interviewers: This interview was conducted by a master's candidate in nursing who has received specialized training in geriatric care, completed courses in qualitative research methods and interview techniques, and possesses relevant clinical experience. On-site support was provided by a nurse with over twenty years of experience in neurology and geriatric nursing (e.g., observing nonverbal cues, clarifying nursing-related questions for the interviewee).

Interview Procedures: Before each interview, rapport was established with participants, who were informed of the study purpose and provided written informed consent. Interviews were audio-recorded, lasted 30–45 minutes per time, and were conducted in a quiet office in the Neurology Department to minimize distractions.​

Data Capture: The first author (interviewer) used active listening techniques (paraphrasing, probing, reflective questioning) to elicit detailed responses. Non-verbal cues (tone, facial expressions, gestures) were documented in field notes and attached to corresponding transcripts.​

### 2.4 Data analysis

#### 2.4.1 Data preparation and management​

**Transcription and annotation:** Within 24 hours of each interview, the first author transcribed audio recordings verbatim, including filler words (e.g., “um”) and pause durations (marked as “[pause: 2 s]”) to preserve contextual meaning. Non-verbal information from field notes was integrated into transcripts as annotations (e.g., “C5: ‘I can’t sleep at night’ [tone: trembling, eyes tearing up]”)—a step aligned with constructivist analysis, which prioritizes holistic interpretation of subjective experiences.​

**Data organization​:** All materials (verbatim transcripts with annotations, field notes) were labeled by participant codes (C1–C19) and interview date, then imported into NVivo 12 software. Two folders were created in NVivo for data management:​

“Raw Data”: Unprocessed transcripts and field notes;​

“Coded Data”: Transcripts with applied codes and thematic labels.​

This structure ensured traceability and efficient access during analysis.​

**Data security maintenance:** Comprehensive data security procedures were implemented across the data lifecycle to protect privacy and prevent breaches: audio recordings were stored on a password-encrypted external hard drive (locked in a secure cabinet, accessible only to the first author and study lead), while digital files (transcripts, NVivo data) were saved to a university secure server with two-factor authentication (2FA), restricted to authorized team members via role-based accounts; a written log tracked data retrieval (date, user, purpose) and was reviewed weekly by the study lead, and data were de-identified with codes (C1–C19), with the de-identification key stored separately in a locked cabinet; data sharing between team members used encrypted email or password-protected files (passwords shared via phone); and post-publication, the external hard drive was physically destroyed by a certified service, digital files were permanently deleted per university policy (5-year retention), and paper materials were cross-cut shredded after the retention period.

#### 2.4.2 Transcript accuracy verification​

Two layers of verification were implemented to ensure transcript accuracy: ① Cross-Verification with Audio: A research assistant (not involved in the original interview) compared 100% of transcripts to audio recordings, flagging discrepancies (e.g., missed words, incorrect tone annotations). Discrepancies were resolved through discussion with the first author until consensus was reached. ② Member Checking: Transcripts of 5 randomly selected participants (C2, C6, C10, C14, C18) were sent to the participants for review. All confirmed the transcripts accurately reflected their statements and emotional expressions, with no revisions requested.​

#### 2.4.3 Research team composition for analysis​

Four team members participated in data analysis, each contributing unique expertise to ensure thematic depth and objectivity: ①Gerontological nursing Nursing Instructor (PhD): Led analysis, leveraging expertise in caregiver mental health and constructivist theory to link codes to contextual meaning (e.g., interpreting “filial guilt” through southwestern China’s cultural framework). ② Neurology Head Nurse ( >20 years of clinical experience): Provided clinical insights, identifying codes related to stroke-specific care challenges (e.g., “difficulty with swallowing feeding”) and ensuring alignment with real-world clinical practice. ③ Geriatric Hospital Supervisor Nurse (10 years of experience): Specialized in non-verbal cue interpretation, validating emotional themes (e.g., “anxiety”) by cross-referencing transcript annotations (e.g., tone changes) to avoid misinterpreting neutral statements. ④ Master’s Candidate in Nursing (no stroke clinical experience): Contributed a “clinically unbiased” perspective, identifying unexpected codes (e.g., “reshaping life meaning”) that might have been overlooked by members with clinical backgrounds.​

#### 2.4.4 Directed content analysis process​

Directed content analysis was conducted following the steps outlined by Hsieh and Shannon (Hsieh & Shannon, 2005), aligned with the study’s research questions (exploring caregiving experiences, emotional states, unmet needs): ① Initial Coding Categories: Key concepts from literature and Maslow’s Hierarchy of Needs were extracted to form initial coding categories (e.g., “care routine management,” “emotional distress,” “unmet professional support”). ② Line-by-Line Coding: Two researchers independently coded transcripts line-by-line, focusing on statements related to caregiver experiences and needs, and categorized data into pre-defined codes. ③ Code Refinement: New codes were assigned to unclassifiable data (e.g., “reshaping life meaning”), and existing codes were iteratively adjusted. ④ Categorization and Thematic Synthesis: Codes were grouped into categories and subcategories based on thematic proximity (e.g., “caregiver burden” as a category, with subcategories of “physical burden” and “emotional burden”). Categories were then synthesized into overarching themes through conceptual analysis. ⑤ Consensus Building: Disagreements in coding or theme identification were resolved through group discussion with the entire research team until consensus was reached.​

#### 2.4.5 NVivo software application​

NVivo 12 was used in the analysis process to enhance rigor and efficiency, with three key functions: ① Code Application: After finalizing the coding scheme, the “coding strip”function linked transcript text segments directly to codes (e.g., tagging “sleep loss” statements to the code “physical burden”). ② Thematic Synthesis Support: The “query” function extracted all text under a specific code (e.g., “unmet training needs”), enabling cross-participant response comparison and pattern identification (e.g., 70% of caregivers mentioned “lack of feeding training”). ③ Data Visualization: The “model” function created a thematic map, illustrating relationships between codes, categories, and overarching themes to track the logic from raw data to final findings.

### 2.5 Ethical considerations

The Ethics Review Committee of Kunming Medical University (KMMU2023MEC194) has reviewed and approved this study to ensure anonymity and privacy protection during data collection and analysis.All records and written materials were retained by one of the authors. Interviews were scheduled at times and locations mutually convenient to the interviewees, who participated on a voluntary basis. Prior to each session, participants were ensured to have understood the study’s objectives, importance, and procedures. As all participants were adults, written informed consent for study participation was obtained from each individual. Participants were also notified that interviews would be audio recorded. To protect privacy, primary caregivers were assigned participant codes (C1–C19).

### 2.6 Trustworthiness

The research team, composed of a full-time nursing instructor, neurology department head nurse, geriatric hospital supervisor nurse, and two graduate students, employed various methods to ensure the trustworthiness of the findings.To address potential influences of team members’ backgrounds on the research process, key characteristics, perspectives, and “stance” of the team are described below:

1. ① Team Lead (Full-time Nursing Instructor): PhD in Nursing with over 20 years of teaching experience in Gerontological nursing. Leveraging familiarity with local values such as filial piety and family-centered care, she refined interview questions (e.g., avoiding suggestive phrasing that implies “family care is a burden”). Based on her prior research on caregivers’ mental health, she focused on the “burden vs. Growth” dynamic while remaining open to emergent themes like positive changes in family bonds.
2. ② Neurology Department Head Nurse: Over 20 years of clinical experience in stroke care. Using frontline experience, she validated the relevance of interview questions (e.g., adding questions about “post-discharge care concerns”), paid attention to doctor-patient power dynamics (avoiding leading questions), and trained interviewers to maintain a neutral stance.
3. ③ Geriatric Hospital Supervisor Nurse: Bachelor’s degree in Nursing with 10 years of geriatric nursing experience. Skilled in interpreting non-verbal cues (e.g., a caregiver’s hesitation may stem from guilt about feeling “inadequate”), she tested the hypothesis that “caregivers often neglect their own needs” through data analysis to prevent bias from affecting theme extraction.
4. ④ Two Graduate Student Assistants (Master’s Candidates in Nursing): Raised in Southwest China, with no clinical experience in stroke care but having completed 60 hours of qualitative research training (including directed content analysis and member checking). They used a “clinically unbiased” fresh perspective to identify unexpected codes (e.g., “reshaping the meaning of life”) and applied NVivo skills to ensure coding consistency. Additionally, they cross-verified interview transcripts to reduce transcription bias.

Interrater Reliability: Two master’s candidates independently coded the first 5 transcripts line-by-line using pre-defined initial coding categories. Post-coding, they held a joint meeting to compare frameworks, flag discrepancies (e.g., one coding “medication management overwhelm” as “physical burden,” the other as “care routine challenge”), and discuss coding rationales. Unresolved discrepancies were mediated by the geriatric nursing instructor (team lead) and neurology head nurse, aligning coding with Maslow’s Hierarchy of Needs (theoretical framework) and participants’ intent. This iterative consensus process was repeated for every subsequent 3 transcripts, with resolved conflicts documented in a “coding discrepancy log” (including date, transcript ID, details, and resolution rationale) for traceability.Member Checking for Credibility: As a key validity measure, 5 randomly selected participants (C2, C6, C10, C14, C18) received interview transcripts (with pseudonyms) and a thematic summary (themes, subcategories, illustrative quotes) via email or in-person meetings. They provided feedback on three aspects: ① transcript content accuracy, ② thematic categorization appropriateness, ③ theme description clarity. C6 clarified their “reluctance to ask neighbors for help” was not just a “social support gap” but tied to “filial piety norms” (fear of being seen as “incompetent”), prompting the team to revise the subcategory to “social support gap influenced by filial norms” and update the thematic description. C14 confirmed the theme “reshaping life meaning” was accurate and suggested adding an example (quitting a part-time job to prioritize care as “family values”), which was integrated to enrich the theme.No other participants requested revisions; all feedback and adjustments were documented in a “member checking log” for transparency.

Upon interviewing the 15th participant, data reached saturation with no new thematic insights emerging. Furthermore, to strengthen data reliability, four additional participants were purposefully recruited, resulting in a final sample of 19 caregivers included in the study.

## 3 Findings

### 3.1 Participant's demographic information

Nineteen respondents were included in this study. The mean age of the respondents was 49.26 ± 11.63 years old. For daily care time, the mean ± standard deviation was 9 ± 4.52 hours, with a range of 4 to 18 hours. Among the 19 caregivers, there were 11 males and 8 females. Regarding characteristics relevant to the study’s purpose: ① Caregiver-patient relationship: 3 pairs were mother-son, 5 pairs were father-son, 2 pairs were father-daughter, 4 pairs were mother-daughter, 3 pairs were spouses, 1 pair was grandparent-grandchild, and 1 pair was father-in-law/son-in-law; ② Informal caregiving duration: The average duration of being an informal caregiver was 3.2 ± 1.5 years; ③ Solo caregiver status: 7 caregivers were solo caregivers, solely responsible for the stroke patients’ daily care without assistance from other family members.Six patients held urban household registration,while thirteen held rural registration.Information about the patients is shown in Table I. Most of the caregivers were the patients’ children, other specific characteristics of the caregivers are shown in Table II. Through the data analysis, three themes and twelve subthemes were generated.

**Table I.** Demographic characteristics of the stroke patients (*n* = 19).

N	Sex	Age (years)	Residence
P1	Female	72	Country
P2	Male	88	City
P3	Male	70	Country
P4	Male	75	Country
P5	Male	75	City
P6	Male	81	Country
P7	Female	67	Country
P8	Female	87	City
P9	Male	80	Country
P10	Male	85	Country
P11	Female	66	Country
P12	Female	74	City
P13	Male	68	Country
P14	Male	86	City
P15	Female	65	Country
P16	Female	65	Country
P17	Male	67	Country
P18	Male	80	City
P19	Female	78	Country

**Table II.** Demographic characteristics of the caregivers (*n* = 19).

N	Sex	Age (years)	Marital status	Residence	Relationship with patients	Education level	Occupation	daily caregiving hours (h)
C1	Male	35	Married	City	mother and son	Master’s degree	Full time	6−8
C2	Male	60	Married	City	father and son	Associate degree	Full time	5−7
C3	Female	50	Married	Country	father and daughter	Middle school diploma	Full time	12−14
C4	Male	51	Married	City	father and son	Undergraduate	Full time	10−12
C5	Female	71	Married	City	man and wife	Elementary school diploma	Unemployed	6−8
C6	Male	49	Married	City	father and son	Undergraduate	Full time	8−10
C7	Male	39	Married	City	mother and son	Undergraduate	Full time	8−10
C8	Female	52	Married	City	mother and daughter	Associate degree	Retire	12−14
C9	Male	51	Married	City	father and son	High school diploma	Full time	5−7
C10	Male	22	Single	City	grandparent and grandchild	Undergraduate	Unemployed	18−20
C11	Female	39	Married	Country	mother and daughter	Middle school diploma	Full time	18−20
C12	Female	48	Married	City	mother and daughter	Associate degree	Full time	4−6
C13	Female	42	Married	City	father and daughter	Undergraduate	Full time	6−8
C14	Male	61	Married	City	Father-in-law and son-in-law	High school diploma	Retire	6−8
C15	Female	42	Married	City	mother and daughter	Undergraduate	Unemployed	4−6
C16	Male	70	Married	City	man and wife	Middle school diploma	Retire	10−12
C17	Female	60	Married	City	man and wife	Undergraduate	Retire	18−20
C18	Male	45	Married	City	father and son	Undergraduate	Full time	7−9
C19	Male	49	Married	City	mother and son	Associate degree	Full time	8−10

### 3.2 Themes

**Figure 1.**
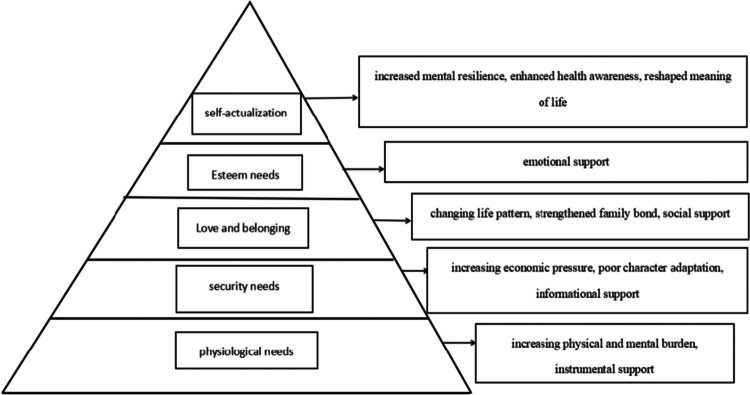
A diagram of different levels of needs for primary care providers based on Maslow's hierarchy of needs.

Most elements of Maslow’s hierarchy of needs can be explained in this study, while some specific codes were derived from caregivers. The data are summarized into three main themes and 12 sub-themes, as shown in Table III. According to Maslow’s hierarchy of needs theory, secondary themes can be divided into physiological needs - increased physical and mental burden, instrumental support, and safety needs - increased economic pressure, maladaptive roles, information support, love and belonging needs, changes in life patterns, strengthening of family bonds, social support, respect needs, emotional support, self-actualization needs, improved mental toughness, enhanced health awareness, and reshaping of life meaning, as shown in Figure 1.

**Table III.** Data analysis result: 3 themes and 12 sub-themes.

Themes	Sub-themes
care-taking challenges and multiple pressures	Increasing physical and mental burden
	Increasing economic pressure
	Poor character adaptation
	Changing life pattern
Caregiver growth and positive changes	Increased mental resilience
	Enhanced health awareness
	Strengthened family bond
	Reshaped meaning of life
Diverse supportive care needs	Instrumental support
	Informational support
	Emotional support
	Social support

#### 3.2.1 Theme 1: care-giving challenges and multiple stresses

Caring for older stroke patients places caregivers under physical and mental stress, financial burden, role adjustment difficulties, and disrupted life patterns, highlighting the multiple challenges and sacrifices they face when coping with stress.

**Subtheme 1: increased physical and mental burden:** Caregiving imposes severe physical and mental strain on individuals. The demanding nature of the task disrupts daily rhythms, leading to sleep deprivation. As one caregiver described: *“I only sleep two hours a day, and I haven’t slept well taking care of him.”* (C10), and loss of appetite: “*Sometimes I can’t eat. Maybe I’m too tired. I don’t have any appetite.”* (C9) Neglect of personal health is common; a caregiver revealed, “*I have carotid plaque and haven’t taken my prescribed medication for two days since coming to the hospital.”* (C4)​

The caregivers are constantly on the edge. Minor changes in the patient’s condition trigger anxiety, as evidenced by, “*This morning my dad said he wasn’t feeling well again. I was in a bad mood all of a sudden and wondered if it was getting worse.”* (C3) The exhaustion of solo caregiving is overwhelming: “*I feel tired after taking care of the patients these days. It is really difficult to do everything alone.”* (C8), and communication issues with patients further intensify stress: “*It’s hard to talk to him; even his daughter can’t.”* (C17)​

Lack of disease knowledge and feel uncertainty. Caregivers worried about the prognosis; one admitted, “*I heard that the first 20 days are critical, so I am still worried.”* (C12), while another agonized, “*What if my mother isn’t cured?”* (C16). These physical and mental pressures highlight the arduous nature of caregiving.

**Subtheme 2: economic pressure surges:** Caregivers consistently reported significant financial burden, primarily stemming from high hospitalization costs for stroke patients and unclear or low medical reimbursement processes—concerns that were particularly prominent among caregivers of rural patients. Multiple participants explicitly mentioned worries about insurance coverage limitations and uncertainty regarding reimbursement amounts, directly reflecting their experience of economic pressure.

*“Money is still a bit of a concern. older adults do have rural health insurance, but I don’t know how much they can apply here.”* (C11)

*“My mother is registered in a rural area, so the medical reimbursement rate may not be very high. I don’t know how much I have been treated.”* (C7)

Some caregivers also noted that cross-regional treatment further complicated reimbursement procedures, adding to their financial stress: “*We came from another county for treatment, and now we have to go back and forth to handle reimbursement—it’s not just the money, but the time spent also adds to the burden.”* (C3)

This economic pressure—tied to participants’ experiences of insurance limitations and unclear reimbursement—reflects structural features of China’s rural healthcare system, particularly stark urban-rural disparities in basic medical insurance across three key areas: funding, reimbursement, and administration. Per National Healthcare Security Administration data (National Healthcare Security Administration [NHSA], 2024), rural residents’ annual per capita insurance funding (≈960 yuan) was only 60.8% of urban residents’ (1,580 yuan). For inpatient care, rural residents had 55%−65% reimbursement at county hospitals (vs. 70%−80% for urban residents at municipal hospitals), with cross-regional care cutting rural rates by an additional 10%−15% (vs. max 5% for urban residents). Rural insurance also covered fewer stroke-relevant rehabilitation treatments/medications (e.g., 3,000-yuan annual cap for stroke physical therapy vs. 5,000 yuan for urban residents) and imposed heavier cross-regional administrative burdens (3−5 workdays for paper-based rural filings vs. 1 workday for urban online real-time filings). These disparities exacerbate caregivers’ financial and time burdens, unlike contexts with universal or integrated regional insurance that mitigate such stress.

**Subtheme 3: poor character adaptation:** Caregivers often struggle to adapt to their roles because of limited caregiving knowledge, work - life conflicts, and insufficient family support.​

A lack of caregiving expertise leaves them ill-equipped for emergencies. A caregiver’s distress was evident when recalling, “*The old man suddenly fell into a coma, and I felt that my understanding of this aspect was so poor. If I had paid more attention to it, I might not have been so flustered and busy fumbling for what to do(like not knowing whether to call the doctor first or check his breathing).”* (C8)

Balancing work and caregiving is a significant challenge. As one caregiver put it, “*Taking care of older adults can have a big impact on my work. My energy is limited, I have to ask for leave and coordinate various things. Sometimes it is really difficult to arrange the time.”* (C7) The constant need to juggle responsibilities strains their ability to maintain both roles.

At home, caregivers face additional burden. “*I have to do a lot of housework, such as cleaning and children’s affairs. My husband is also unreliable.”* (C13) This lack of support compels difficulties in caregiving. Together, these factors highlight the common issue of poor character adaptation among caregivers.

**Subtheme 4: social isolation:** Caregivers reported significant social isolation, which primarily stemmed from the disruption of their former hobbies, leisure activities, and social interactions due to long-term caregiving responsibilities—representing a key lifestyle change driven by their care role.

*“I usually like to do tai chi—before, I’d go to the community square every morning with a group of friends, but since I started taking care of (the patient), I can’t do it anymore. I haven’t seen those friends for months.”* (C17)

*“Came to the hospital to take care of (the patient), and a few old friends used to call me to go out—like to visit the park or have tea. But now I never get the chance; I have to stay with (the patient) all day. Slowly, we don’t keep in touch as much.”* (C16)

#### 3.2.2 Theme 2: caregiving growth and positive change

Caregivers experience increased mental resilience, health awareness, strengthened family bonds, and a renewed sense of meaning in life while caring for patients.

**Subtheme 1: increased mental resilience:** In caregiving, individuals gradually adapt, showing patience, persistence, and a strong sense of responsibility, along with increased empathy and a positive outlook on the patient’s future.Patience is key when facing daily caregiving challenges. One caregiver’s experience administering medication illustrates this well: *“When he takes the medicine, he can’t swallow it, so he has to give it several times each time, and if it doesn’t go in, he will give it two times. Until it goes down.”* (C10) Their determination, despite difficulties, shows growing patience.​

Caregivers also developed a deep-seated sense of responsibility. As stated, *“Now it is my responsibility and obligation to take care of the patients in the hospital. I just want to do my best to take care of older adults and deal with other things as they come.”* (C2), indicating that they internalized caregiving as both a duty and commitment.​

Empathy shapes the caregiver-patient bond. When a caregiver’s mother worried about recovery, the response, “*My mother is always asking me, ‘Is this going to be good, is it going to be good, if only it were.’ I would reassure her and tell her to relax and take it slow.”* (C12) This shows the caregiver’s ability to recognize her mother’s anxiety and proactively use specific, encouraging details to ease her worries, rather than just offering general comfort—directly reflecting the empathy gained through caregiving.

Improvements in the patien’s condition boosted caregivers’ positivity. “*The patient’s condition has been improving these days, the patient is getting better, then I am in a good mood, and I have more confidence in the patient’s recovery.”* (C1) This positive shift benefits both the caregiver and patient.​

**Subtheme 2: increased health awareness:** By taking care of patients and observing their situation of patients around them, caregivers realize the importance of health and hope to keep healthy by adjusting their lifestyle.

*“Coming to the hospital to see these patients, my heart is still a little touched, so I still need to control my diet and exercise.”* (C19)

*“I think I should take good care of my body while I am young, otherwise it would be too painful to be ill.”* (C6)

**Subtheme 3: family bond strengthening:** When a patient is hospitalized amid family adversity and stress, some caregivers’ family networks grow notably closer, manifesting in four key dimensions: interdependence (emotional and practical mutual support), cooperative division of labor (task allocation by strengths), adaptability (adjusting life/work for care), and shared positive mentality (uniting for patient recovery)—all deepening family emotional bonds.

Cooperative division of labor eases individual burden and fosters mutual support. As one caregiver noted, “*We change shifts as a family—my brother does morning rehab with Dad, I handle afternoon meals/medicine, and my sister chats with him at night. Splitting work keeps us less tired and not alone.”* (C13) This allocation boosts efficiency and shared responsibility in recovery.

Interdependence and positive mentality also appear in cherished family time. Another caregiver shared, “*Before Dad’s hospitalization, we were busy separately—I worked out of town, brother ran a shop, only meeting on holidays. Now we’re all back to care for him. After shifts, we chat about Dad’s past in the hospital corridor; we’ve never been closer.”* (C8) This shift from individual busyness to united care reflects emotional reliance and prioritized family bonds, reinforcing optimism to overcome hardships.

**Subtheme 4: reinventing the meaning of life:** Caring for patients drives caregivers to deeply reflect on life’s meaning, fostering a unique perspective that includes future foresight, acceptance of life uncertainties, and an open-minded view of life’s natural cycle.​

Regarding future planning, a caregiver’s insight, “*In our generation, there are not many sisters to take care of older adults, and it is the turn of our children, who are the only child.”* (C14) reveals how caregiving prompts the contemplation of long-term family support dynamics and the challenges future generations may face.​

Caregivers also learn to accept the unpredictability of life. “*Human affairs are indefinable, and what will happen tomorrow nobody knows.”* (C2) encapsulates their realization that the caregiving journey, filled with unexpected events, teaches them to approach life with resilience.​

Finally, they adopt an equanimous attitude towards life, old age, illness, and death. “*I take life very lightly. I think birth, old age, illness and death are normal.”* (C19) shows how caregiving broadens their perspective, allowing them to find peace in accepting life’s inevitable phases.

#### 3.2.3 Theme 3: diverse supportive care needs

Caregiver needs are diverse and focus on instrumental support such as medical and in-home care alternatives; informational support, including disease knowledge and rehabilitation guidance; emotional support; seeking empathy and respect; and social support, involving family, community, and policy assistance.

**Subtheme 1: instrumental support:** Most caregivers urgently require multi-faceted instrumental support, particularly in the areas of medical care and daily living care. In terms of medical care, the core needs of caregivers focus on obtaining continuing care guidance from nurses and receiving professional medical advice—they rely on nurses’ expertise to address uncertainties about post-hospital care and long-term treatment.

In continuing care, caregivers are often unsure about post-hospital care. As one caregiver worried, “*I don’t know how I’m going to take care of my mom after I leave the hospital, like how I’m going to exercise, what I’m going to watch out for, what I’m going to eat.”* (C3) Uncertainty about long-term treatment also troubles them, with another saying, “*Now I'm just worried about how long it will take him to recover completely if he takes this medicine, or if he has to take this medicine for life—these are things I want to ask the nurse clearly, but I always forget to bring them up during rounds.”* (C11) These statements directly reflect caregivers’ need for nurses to provide systematic continuing care and medical guidance.

In living care, caregivers seek help from temporary and regular services. “*I definitely want someone to help me if I can. I just want a good caregiver or a relative to help me.”* (C4) reveals their physical burden, while *“I still need someone to help me when I’m in a bad mood.”* (C17) emphasizes emotional strain. These results show that multi-faceted instrumental support is essential for caregivers to handle their roles.

**Subtheme 2: information support:** Caregivers’ need for information support is prominently manifested in their yearning for disease knowledge, proactive pursuit of information, reliance on professional guidance, and concerns regarding future rehabilitation plans.​

The lack of disease-related knowledge often leaves caregivers anxious and confused. As one caregiver lamented, “*I don’t know much about the disease, and I don’t know how serious my mother is now.”* (C11)

In their quest for better caregiving, caregivers actively seek professional guidance, especially regarding future rehabilitation strategies. A caregiver expressed a strong desire to learn, stating, “*I want to ask the doctors and nurses about what to do in the later stage of rehabilitation.”* (C4)

**Subtheme 3: emotional support:** Caregivers’ emotional support needs varied significantly. Some find comfort in medical services; a caregiver noted, “*The service attitude of doctors and nurses is quite good, and it feels good.”* (C3), indicating that positive interactions with the medical staff can provide emotional relief.​

However, many caregivers were long enough to empathize. One caregiver’s past experience, “*Seeing him in the hospital reminds me of when I was in the hospital myself, when the old man didn’t even come to take care of me.”* (C5), reveals personal vulnerabilities that heighten their need for understanding. Additionally, lack of warmth from healthcare providers causes frustration. Another caregiver said, “*Some doctors give people the impression that they are so cold, I wish they could put themselves in their shoes sometimes!”* (C8), emphasizing the importance of empathetic engagement in meeting the caregivers’ emotional needs.

**Subtheme 4: social support:** This study revealed that caregivers’ social support needs to span multiple aspects, including family, patient community, and policy assistance, while some also face shortages in support from friends and medical services.​

In terms of family support, caregivers often rely on relatives with relevant expertise. As one caregiver shared, “*My son’s mother-in-law is also a nurse in the hospital, and we ask her when we have questions about caring for patients.”* (C5), demonstrating how family connections offer practical help. The patient community serves as a valuable support network. Caregivers in the same ward often engage in mutual aid; for instance, “*The family members in our ward will help each other. For example, they will take care of older adults for me when I have something to do. I will also help them when they have something to do with their family.”* (C7)

However, policy support currently falls short of this. A caregiver pointed out, “*older adults have medical insurance, but the reimbursement rate is not high, so the cost of hospitalization is still a bit high for us.”* (C6), highlighting financial strain due to insufficient policy benefits. Support from friends is also limited, as stated, “*Friends have their own things going on, so it’s not easy to ask them for anything, and it’s not realistic to ask other people to take care of them.”* (C7) In addition, caregivers hope for more help from the medical services. One expressed, “*It would be nice if the hospital could give us more help, like someone to guide us when we have tests.”* (C1), indicating a need for enhanced medical guidance and support.

## 4 Discussion

This study explores the experiences and needs of caregivers of elderly stroke patients, identifying three overarching themes:“Caregiving Challenges and Multiple Stresses,” “Caregiving Growth and Positive Change,” and “Diverse Supportive Care Needs.” These themes align with Maslow’s Hierarchy of Needs theory, where sub-themes map to distinct motivational levels (e.g., Physiological Needs: physical/mental burden, instrumental support; Safety Needs: economic pressure, role adaptation; love and belonging: lifestyle disruption, family bonding; Respect Needs: emotional support; self-actualization: resilience, health awareness, life meaning). This theoretical framework underscores the multi-faceted nature of caregivers’ struggle and growth.

### 4.1 Caregiving challenges and multiple stresses: addressing basic needs in Maslow’s hierarchy

The theme of caregiving challenges is directly related to the Physiological and Safety Needs in Maslow’s model. Caregivers’ physical and mental exhaustion (e.g., sleep deprivation, neglect of personal health; C4, C9, C10), coupled with economic pressures (e.g., low rural medical reimbursement rates; C7, C11), reflects unmet basic survival needs. Role adaptation difficulties (e.g., work-care conflicts; C7) and lack of disease knowledge (e.g., uncertainty about post-hospital care; C3) further undermine their sense of safety and security.In Chinese culture, filial piety reinforces caregivers’ duty to prioritize parental care, often at the expense of their own needs. As noted, child caregivers exhibit profound sacrifice driven by the Confucian value of respecting and supporting aging parents (Dong et al., 2014). This cultural mandate, combined with the tangible economic strain of urban-rural medical insurance disparities, exacerbates rural caregivers’ burden: for example, the average annual rehabilitation cost for post-stroke patients in China is approximately 25,000 yuan. Based on 2023 data, rural caregivers face out-of-pocket expenses of ~10,000 yuan (43.5% of rural residents’ per capita disposable income, ~23,000 yuan) after 60% insurance reimbursement, while urban caregivers only pay ~5,000 yuan (10.2% of urban residents’ per capita disposable income, ~49,000 yuan). This 5,000-yuan gap in out-of-pocket costs directly explains why rural caregivers in this study (e.g., C7, C11) repeatedly expressed anxiety about reimbursement—far more than urban caregivers.

Unlike the norm in Eastern countries where female caregivers usually predominate and report higher burdens (Dong et al., 2014), this study (*n* = 19) included 11 male caregivers (57.9%) and 8 female caregivers (42.1%), with males slightly outnumbering females. Though the small sample size limits the generalizability of this gender distribution (and it is not interpreted as representative of Eastern stroke caregivers overall), the finding is noteworthy: it deviates from the “female-dominant” demographic common in existing stroke caregiver research (Swinkels et al., 2019), prompting potential inquiry into whether contextual factors (e.g., rural southwestern China, hospital-based recruitment) shape caregiver gender composition, and it highlights male caregivers—a group often underrepresented in caregiving studies (Dong et al., 2014) but likely facing unique stressors and using distinct coping strategies, thus meriting attention. As existing literature notes (Swinkels et al., 2019), female caregivers typically bear heavier burdens due to stronger emotional engagement with care recipients, societal expectations of women as “primary nurturers,” and greater challenges balancing caregiving with domestic or professional roles; in contrast, male caregivers in this study rarely described emotional distress, instead emphasizing “finding practical solutions” (e.g., consulting doctors directly for medical guidance) or “not wanting to express stress to others” (adhering to the principle emphasized in traditional Chinese culture that men should be steadfast and unyielding.). Importantly, this difference in stress expression does not mean male caregivers experienced less stress—their stress may have manifested in less visible forms (e.g., reduced work hours, unspoken frustration) not fully captured in the analysis, which aligns with the notion that gender differences in caregiving burden often relate to expression rather than absence (Swinkels et al., 2019).

This cultural mandate of filial piety aligns with the “Love and Belonging” dimension of Maslow’s hierarchy, as family responsibility becomes a core social bond. However, this commitment also intensifies stress: caregivers report worry, frustration, and uncertainty about patients’ prognosis (Woodford et al., 2018; Li et al., 2024), echoing findings that unmet physiological and safety needs exacerbate psychological burden. To address these needs, medical staff should implement family-centered psychological education interventions to enhance caregivers’ coping skills, as psychological nursing has been shown to reduce caregiver stress (Mou et al., 2022).

### 4.2 Caregiving growth and positive change: fulfilling higher-order needs

The theme of caregiving growth corresponds to Self-Actualization Needs in Maslow’s hierarchy, where caregivers derive meaning from adversity. Sub-themes such as increased mental resilience (e.g., patience in medication administration; C10), health awareness (e.g., diet and exercise focus; C6, C19), and strengthened family bonds (e.g., collaborative caregiving; C13) reflect personal growth and emotional adaptation.The Chinese cultural emphasis on family harmony (Confucian values) fosters collective resilience. Caregivers often transform challenges into opportunities for positive family dynamics, such as interdependent caregiving roles and shared responsibility (Chen & Fan, 2010; Tan et al., 2024). For example, family members in this study adopted shift-based care to reduce individual burden (C13), illustrating how adversity strengthens social cohesion support this growth, and how healthcare providers can use cognitive-behavioral therapy or narrative therapy to help caregivers reframe negative perceptions. Community-based support groups, in which caregivers share success stories, can enhance confidence in caregiving and recovery (Viens et al., 2024). At the family level, promoting open communication and mutual support further solidifies caregiving networks.

### 4.3 Diverse supportive care needs: bridging gaps across hierarchy levels

The theme of supportive care needs spans all levels of Maslow’s hierarchy, from instrumental support (e.g., post-hospital care guidance; C3) for physiological safety to emotional support (e.g., empathy from medical staff; C8) for respect and belonging. Caregivers’ urgent requests for disease knowledge (C11) and rehabilitation guidance (C4) highlight unmet informational needs, while social support gaps (e.g., limited friend involvement; C7) underscore the importance of community and policy interventions.To address these needs, hospitals should provide personalized discharge care plans (e.g., exercise, diet, medication guidelines) and offer long-term care insurance consultations to reduce financial stress. Community-level initiatives, such as multidisciplinary extended care teams and intergenerational activities, can alleviate family burden and promote social integration (Sidek et al., 2022;Hu et al., 2023). Emotional support from the medical staff, including empathetic communication and clear information delivery, is equally critical for meeting caregivers’ respect needs (C3, C8).

## 5 Recommendation

This study identified caregivers of elderly stroke patients have multi-dimensional needs (care knowledge/skills, home rehabilitation guidance, economic, emotional, social support) (Viens et al., 2024). To address these comprehensively, systematic interventions across micro (individual), meso (organizational/community), and macro (societal/policy) levels are proposed as follows.

### 5.1 Micro-level: individual caregiver support

Knowledge & skill support: Establish regular communication channels (e.g., bilingual/dialect pictorial discharge manuals for low-literacy caregivers) and conduct longitudinal needs assessments (Chinese version of Caregiver Needs Inventory) at 1-week/1-month/3-month post-discharge to adjust guidance dynamically.

Emotional support: Integrate family-centered values (e.g., 2-weekly family care planning check-ins via WeChat/in-person) to share burdens; nurses follow up individually via WeChat/WhatsApp (Sidek et al., 2022) for one-on-one problem-solving (e.g., feeding difficulties).

### 5.2 Meso-level: organizational & community interventions

Healthcare institutions: Develop multilingual telehealth portals (real-time consultation, test reminders) and assign ward “caregiver navigators” to accompany tests/explain results; ease operational burdens.

Communities: Train nurses in stroke care (pressure ulcer prevention, swallowing monitoring), build hospital-community telemedicine links (doxy.me), launch EHR-integrated home service apps (vital sign logging/alerts), and set up peer support groups (WeChat workshops) for practical/emotional exchange.

### 5.3 Macro-level: societal & policy changes

Policy optimization: Expand long-term care insurance (LTCI) to cover post-stroke rehab, assign hospital insurance coordinators for disabled patients; promote nationwide cross-provincial reimbursement real-time settlement, add rural caregiver allowances and narrow urban-rural stroke expense reimbursement gaps.

Societal awareness: Launch public campaigns (CCTV/WeChat) to highlight caregiver needs; encourage enterprises to offer flexible working hours for caregivers.

## 6 Limitations

This study had two main limitations. First, most of the interviewees were children. While representativeness is not the main concern in qualitative research, this narrow focus might have failed to fully answer the research question, missing perspectives from patients, other family members, or professional caregivers. Second, the study was conducted only in a tertiary first-class hospital, the highest-level medical institution in China known for advanced technology, comprehensive disciplines, and strong research. The unique culture, resources, and patient mix of this single hospital may have affected the results, limiting their applicability in other settings. Future research should expand the participant pool and conduct multicenter studies across different hospital levels and regions to obtain broader insights.

## 7 Conclusions

This study revealed the stress and growth of primary caregivers for elderly stroke patients, highlighting their need to align with Maslow’s Hierarchy of Needs. These include physiological burdens, safety-related economic and informational needs, love and belonging requirements from social and family aspects, respect in the form of emotional support, and self-actualization shown as mental toughness and meaning-seeking. Medical staff should focus on caregivers’ experiences, leveraging Maslow’s Hierarchy as a framework. By providing instrumental support for physiological relief, information for safety, and emotional support for respect, they can enhance caregivers’ potential and care service quality. China should prioritize caregivers’ feelings and growth-oriented self-actualization needs. Targeted interventions based on Maslow’s hierarchy can effectively support caregivers.

## Author contributions

CRediT: **Haiyan Wang:** Conceptualization, Methodology, Data Curation, Writing – review & editing, Supervision, Project administration, Funding acquisition; **Xuejuan Yang:** Methodology, Investigation, Data Curation, Writing-original draft, Writing – review & editing, Supervision; **Siying Chen:** Writing – review & editing, Visualization, Supervision; **Yin He:** Writing – review & editing, Supervision, Project administration; **Yuying Chen:** Methodology, Supervision, Writing – review & editing.

## Data Availability

The datasets generated during and/or analyzed during the current study are available from the corresponding author upon reasonable request.
